# Automated chart review utilizing natural language processing algorithm for asthma predictive index

**DOI:** 10.1186/s12890-018-0593-9

**Published:** 2018-02-13

**Authors:** Harsheen Kaur, Sunghwan Sohn, Chung-Il Wi, Euijung Ryu, Miguel A. Park, Kay Bachman, Hirohito Kita, Ivana Croghan, Jose A. Castro-Rodriguez, Gretchen A. Voge, Hongfang Liu, Young J. Juhn

**Affiliations:** 1 Department of Pediatric and Adolescent Medicine, Mayo Clinic, 200 1st Street SW, Rochester, MN 55905 USA; 2 Asthma Epidemiology Research Unit, Mayo Clinic, Rochester, MN USA; 3 Departement of Pediatrics, University of New Mexico, Albuquerque, NM USA; 4 Division of Biomedical Statistics and Informatics, Mayo Clinic, 200 1st Street SW, Rochester, MN 55905 USA; 5 Division of Allergic Disease, Mayo Clinic, Rochester, MN USA; 6 Department of Medicine Research, Mayo Clinic, Rochester, MN USA; 7 Division of Pediatrics, School of Medicine, Pontificia Universidad Catolica de Chile, Santiago, Chile; 8 Division of Neonatology, Children’s Hospitals and Clinics of Minnesota, Minneapolis, MN USA

**Keywords:** Asthma, API, Epidemiology, Informatics, NLP

## Abstract

**Background:**

Thus far, no algorithms have been developed to automatically extract patients who meet Asthma Predictive Index (API) criteria from the Electronic health records (EHR) yet. Our objective is to develop and validate a natural language processing (NLP) algorithm to identify patients that meet API criteria.

**Methods:**

This is a cross-sectional study nested in a birth cohort study in Olmsted County, MN. Asthma status ascertained by manual chart review based on API criteria served as gold standard. NLP-API was developed on a training cohort (*n* = 87) and validated on a test cohort (*n* = 427). Criterion validity was measured by sensitivity, specificity, positive predictive value and negative predictive value of the NLP algorithm against manual chart review for asthma status. Construct validity was determined by associations of asthma status defined by NLP-API with known risk factors for asthma.

**Results:**

Among the eligible 427 subjects of the test cohort, 48% were males and 74% were White. Median age was 5.3 years (interquartile range 3.6–6.8). 35 (8%) had a history of asthma by NLP-API vs. 36 (8%) by abstractor with 31 by both approaches. NLP-API predicted asthma status with sensitivity 86%, specificity 98%, positive predictive value 88%, negative predictive value 98%. Asthma status by both NLP and manual chart review were significantly associated with the known asthma risk factors, such as history of allergic rhinitis, eczema, family history of asthma, and maternal history of smoking during pregnancy (*p* value < 0.05). Maternal smoking [odds ratio: 4.4, 95% confidence interval 1.8–10.7] was associated with asthma status determined by NLP-API and abstractor, and the effect sizes were similar between the reviews with 4.4 vs 4.2 respectively.

**Conclusion:**

NLP-API was able to ascertain asthma status in children mining from EHR and has a potential to enhance asthma care and research through population management and large-scale studies when identifying children who meet API criteria.

## Background

According to a report from the Agency for Healthcare Research and Quality, asthma is one of the five most burdensome diseases in the United States [1]. Asthma is the most common chronic illness in childhood, affecting 4–17% of children in the United States [2] and 2.8–37% of children worldwide depending on countries [3] with significant healthcare, social, and academic burden. [4, 5] Despite the availability of evidence-based guidelines for asthma management and effective asthma therapies, there has been virtually no change between 1990 and 2010 in years lived with asthma-related morbidity in the United States [2].

One of the major challenges in the current asthma research is inconsistency in study results reported in genome-wide association studies [6], clinical trials [7, 8], and studies addressing heterogeneity of asthma [9, 10], making it difficult to apply these results to clinical practice and advancement of the field. Apart from the true biological heterogeneity of asthma, other important sources of variability in the above studies include inconsistent asthma criteria (physician diagnosis vs. subjective determination based on diverse asthma criteria) and ascertainment processes (chart review vs. surveys), which may obscure a better understanding of biological heterogeneity of asthma. As an example, literature showed asthma status according to parental report was associated with significant misclassification bias [11], and studies including younger children (e.g., < 3 years old) for whom asthma diagnosis rarely occur may not use physician diagnosis nor International Classification of Diseases (ICD) codes for asthma. The latter is very important for many reasons. First, the burden of disease is greatest in preschoolers with a significantly higher proportion of emergency department visits, more hospitalizations, more sleep disturbances, and more limitation of family activities/play than older children [12, 13]. Second, the irreversible impairment in lung function may occur during the preschool period, suggesting a window of opportunity to perhaps prevent irreversible damage [14]. It is possible that the repeated and cumulative lung injury caused by various respiratory infections that are frequent at this age may be causal or important intercurrent factors affecting lung growth and asthma persistence. Despite these limitations, these approaches are still frequently used for large-scale asthma studies. While implementing laboratory tests can be considered, it can be impractical for studies based on large cohorts.

Until today, manual chart review is the most accurate method to identify asthma cases regardless of physician diagnosis of asthma, but this becomes a challenge for large-scale studies. There is an emerging need for developing a medical informatics approach like natural language processing (NLP) that processes free-text and classifies asthma status at a patient level in the era of electronic health records (EHR).

In the medical community, Asthma Predictive Index (API) [15] is a validated criterion and can be a potential option for asthma studies and help to reduce the variability described above in the future research work for asthma in children. We recently demonstrated feasibility of using NLP algorithms for an existing other asthma criteria, Predetermined Asthma Criteria [16–18], which was originally developed by Yunginger et al. [19] and has been used extensively in research for asthma epidemiology [20, 21]. At present, given the potential suitability of API to a retrospective study [22, 23], the recommended use of API by National Asthma Education and Prevention Program guidelines [24], and unavailability of NLP algorithm for API, developing and validating an NLP-based API algorithm would be worthwhile.

To date, there is no NLP algorithm enabling automated chart review for EHR to ascertain asthma status in children based on API. Therefore, the main aim of this study was to develop and validate an NLP algorithm to identify patients that meet API positive criteria by assessing criterion and construct validity in a retrospective study.

## Methods

The study was approved by the institutional review boards at the Mayo Clinic and Olmsted Medical Center, located in Olmsted County, Minnesota.

### Study setting

Demographic characteristics of the population of Rochester and Olmsted County were similar to those of the U.S. Caucasian population, with the exception of a higher proportion of the working population of this community being employed in the health care industry [19]. Olmsted County has a few important epidemiological advantages for conducting retrospective studies such as this because medical care is virtually self-contained within the community. In addition, research authorization for using medical records for research purposes is obtained from the patients the first time they ever register with a provider in the community. The rate of granting this authorization is about 95% in Olmsted County [25]. Once this permission is granted, each patient is assigned a unique identifier under the auspices of the Rochester Epidemiology Project, which has been continuously funded by the National Institute of Health (NIH) since 1966 [26]. Using this unique identifier, all clinical diagnoses and events, and detailed information from every interaction among the patients and providers are retrieved from detailed patient-based medical records [26]. As this resource has been electronically available since 1997 (i.e., the inception of the EHR at Mayo Clinic), it enables us to retrieve all asthma-related events and associated free-text information (e.g., symptoms, visits, and medications) electronically to ascertain asthma status based on API [15].

### Study design

This is a cross-sectional study nested in a birth cohort study, which was designed to develop and validate an NLP algorithm for ascertaining asthma status by API (NLP-API) using convenience samples. The NLP algorithm was developed on the training cohort and evaluated on an independent test cohort for which asthma status by manual chart review (CW) was already available based on API. Criterion validity was assessed by determining concordance of asthma status by API between NLP-API and manual chart review. Construct validity of NLP-API was assessed by determining the association between asthma status ascertained by NLP algorithms and the known risk factors for asthma.

### Study subjects

There were two cohorts enrolled in this study. The first cohort was used to develop the NLP algorithm (i.e., training cohort, *n* = 87) and the second one was used for validating the results (i.e., test cohort, *n* = 427). The training cohort was made up of subjects who were all born after the implementation of the EHR at the Mayo Clinic (i.e.1998–2002) [16]. Briefly, the training cohort were children who were enrolled in the Mayo Clinic sick child care program and their parents agreed to participate in a previous study assessing factors associated with parents’ care-seeking behavior for mild acute illness of young children. Of the original 115 children, subjects were excluded due to the following reasons: 1) change of research authorization status (*n* = 3), 2) adopted children (*n* = 4; one of the major criteria of API is parental history of asthma), and 3) primary care at a non-Mayo site (*n* = 21; NLP was only available for Mayo EHR during the study period).

The validation part of the study utilized a random sample of the 2002–2006 population-based birth cohort who had been enrolled in a previous asthma study and had medical records mainly at Mayo Clinic, Rochester, Minnesota [27]. Briefly, the original study enrolled 579 subjects comprised of 282 late-preterm infants (34 0/7 to 36 6/7 weeks of gestation) and 297 gender- and birth year-matched term infants (37 0/7 to 40 6/7 weeks of gestation) randomly selected from the 2002–2006 birth cohort born in Olmsted County, Minnesota. In this study, a total of 152 subjects were excluded due to the following reasons: 1) change in research authorization status (*n* = 17), 2) adopted children (*n* = 3), and 3) primary care outside Mayo Clinic (*n* = 132), leaving 427 study subjects for this present study.

### Asthma predictive index (API)

The presence of asthma was defined if frequent wheezing episodes (i.e., two or more wheezing episodes within one year) AND either one of the major criteria or two of the minor criteria are satisfied (Table 1) [15, 22, 23]; otherwise, study subjects were considered non-asthmatics. An index date of asthma was defined as the earliest date when the API criteria were met. The operational procedures for API for retrospective studies were described in a previous study [23].

**Table 1:** Asthma Predictive Index (API) for asthma^a^ ascertainment

Major Criteria	Minor criteria
1. Physician diagnosis of asthma for parents	1. Physician diagnosis of allergic rhinitis for patient
2. Physician diagnosis of eczema for patient	2. Wheezing apart from colds
	3. Eosinophilia (≥ 4%)

^a^Asthma is determined by frequent wheezing episodes (two or more wheezing episodes within one year) plus at least one of major criteria or two of minor criteria

### Development of NLP algorithm for API

**Fig. 1:**
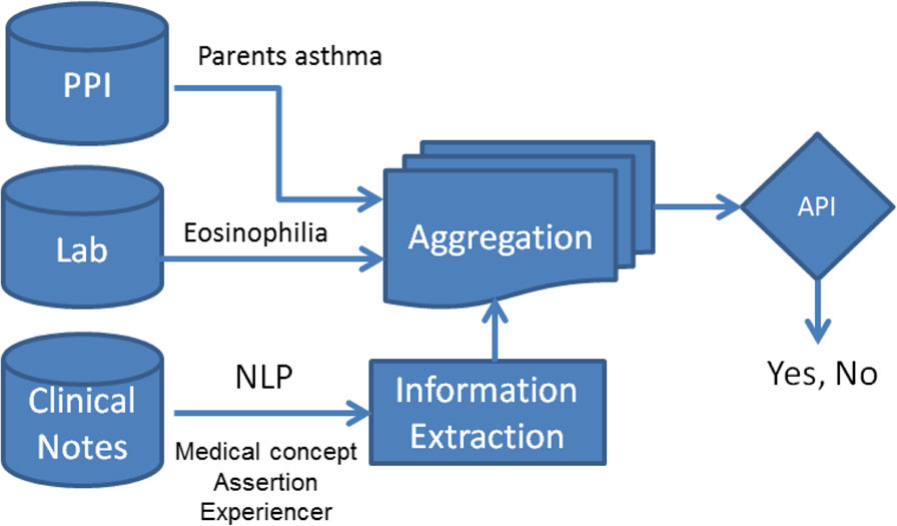
Overview of NLP-API algorithm (Abbreviation: PPI – Patient Provided Information)

The overall process for the NLP-API algorithm to ascertain asthma status is depicted in Fig. 1. Our NLP algorithm used three different sources of data—i.e., clinical notes, laboratory data, and patient-provided information. Parents’ asthma information (one of major criteria) was identified from both clinical notes (family history section of the patient’s chart) and patient-provided information, and an eosinophil value was extracted from the lab data if tested for any reason. The other items of API criteria (i.e., eczema, allergic rhinitis, and wheezing ± colds) were extracted from clinical notes using pattern-based rules, assertion status (e.g., non-negated, associated with patient), and section constraints (e.g., diagnosis section). Then, we developed expert rules implementing API criteria (Table 1). Our algorithm was built in the open-source NLP pipeline MedTagger (https://sourceforge.net/projects/ohnlp/files/MedTagger/) developed by Mayo Clinic [16]. In this pipeline, there are two basic conceptual blocks to identify API-positive asthmatics. 1) A text processing component which finds evidence text in EHRs to match specific API criteria, and 2) A patient classification component which decides the patient’s API status based on available evidence.

### Asthma risk factor variables

These variables were collected during the previous study [27] and utilized for this study to assess construct validity (i.e., association between NLP-API (vs. manual chart review) and asthma risk factors for comparison purpose). These included birth weight, small for gestational age, mode of delivery (Cesarean section vs. vaginal delivery), gestational age, a family history of asthma and other atopic conditions such as allergic rhinitis or atopic dermatitis, a history of patient’s allergic rhinitis or eczema, maternal smoking during pregnancy, passive smoking exposure after birth (up to the first 6 years of life), and breast-feeding history. As all asthmatics did not fulfill the same criteria, we also assessed individual items included in API as risk factors for determining construct validity.

### Statistical analysis

Performance of NLP-API was assessed for both criterion and construct validity. For criterion validity, we calculated agreement rate, Kappa index, and validation index (sensitivity, specificity, positive predictive value, and negative predictive value) for concordance in asthma status between NLP-API and manual chart review as gold standard. Using logistic regression models, construct validity was assessed by determining the association of asthma status ascertained by NLP-API with the known risk factors for asthma as NLP-API is expected to be correlated with the known risk factors if NLP algorithm reflects true asthma. The associations were summarized by odds ratios and their corresponding 95% confidence intervals. All analyses were performed using JMP statistical software package (Ver 10; SAS Institute, Inc., Cary, NC).

## Results

### Study subjects

Characteristics of the test cohort are summarized in Table 2. 209 (48%) were male, and 315 (74%) were female. The median age (interquartile range) at the last follow-up date was 5.3 years (3.6, 6.7). 36 (8%) met the API by manual chart review, and 39 (9%) and 102 (24) had history of allergic rhinitis and eczema by physician diagnosis during the study period.

**Table 2:** Demographics of the test cohort

	Test cohort (*n* = 427)
Age at the last follow-up date, years, median (interquartile range)	5.3 (3.6, 6.7)
Male, n (%)	209 (48%)
White, n (%)	315 (74%)
Asthma (ascertained by abstractors), n (%)	36 (8%)
Allergic rhinitis, n (%)	39 (9%)
Eczema, n (%)	102 (24%)
Family history of asthma, n (%)	101 (23%)
Maternal smoking during pregnancy, n (%)	33 (7%)
History of breastfeeding, n (%)	354 (84%)

### Concordance in asthma status between NLP-API and manual chart review (criterion validity)

For the test cohort, Kappa index and agreement for asthma status between NLP algorithm and manual chart review were 0.86 and 97%, respectively. Sensitivity, specificity, positive predictive value, and negative predictive value for NLP algorithm in predicting asthma status were 86%, 98%, 88%, and 98%, respectively. Overall, these results were similar with regard to gender, ethnicity and gestational age (Table 3).

**Table 3:** Agreement of asthma ascertainment between NLP and manual chart review (criterion validity)

Test cohort (*n* = 427)	Kappa-index	Overall agreement rate	Sensitivity	Specificity	PPV^a^	NPV^b^
Overall	0.86	97%	86%	98%	88%	98%
Sex						
Male (*n* = 209)	0.89	98%	90%	98%	90%	98%
Female (*n* = 218)	0.82	97%	80%	99%	85%	98%
Race						
Caucasian (*n* = 315)	0.83	97%	81%	98%	88%	98%
Non-Caucasian (*n* = 106)	0.94	99%	100%	98%	90%	100%
Gestational age						
Late Preterm (*n* = 197)	0.82	96%	84%	98%	84%	98%
Term (*n* = 230)	0.90	98%	88%	99%	93%	99%

^a^PPV: Positive Predictive Value

^b^NPV: Negative Predictive Value

### Association of asthma status of NLP-API with the known risk factors (construct validity)

The results for construct validity are summarized in Table 4. A correlation analysis of each of the risk factors with asthma status by NLP and manual chart review was run on the test cohort independently. Asthma status by both NLP and manual chart review were significantly associated with the known asthma risk factors. For example, children with asthma compared to those without asthma had higher odds for having a history of allergic rhinitis, eczema, family history of asthma, and maternal history of smoking during pregnancy (*p* value < 0.05). For the factors of family history of other atopic conditions, passive smoking exposure, gestational age, birth weight, childcare attendance, and breastfeeding history, the direction and effect sizes were comparable between manual chart review and NLP-API, although the associations were not statistically significant.

**Table 4:** Associations of asthma status determined by NLP and manual chart review with known risk factors for asthma (construct validity)

	By NLP				By manual chart review			
	No asthma (*n* = 392)	Asthma (*n* = 35)	OR^d^ (95% CI)	*p*-value	No asthma (*n* = 391)	Asthma (*n* = 36)	OR^d^ (95% CI)	*p*-value
Age,^a^ years, median (IQR)	5.2 (3.4, 6.7)	6.2 (4.6, 6.8)	1.2 (1.0, 1.4)	.01	5.1 (3.5, 6.7)	6.3 (4.4, 6.8)	1.2 (1.0, 1.4)	.02
Male, *n* (%)	188 (47%)	21 (60%)	1.6 (0.8, 3.2)	.17	188 (48%)	21 (58%)	1.5 (0.7, 3.0)	.23
White, *n* (%)	290 (75%)	25 (71%)	0.8 (0.3, 1.7)	.62	288 (75%)	27 (75%)	1.0 (0.4, 2.2)	.97
Birth weight, median (IQR)	3.14 (2.5,3.5)	2.8 (2.3,3.4)	0.9 (0.9, 1.0)	.08	3.1 (2.5–3.5)	2.8 (2.4–3.4)	0.9 (0.9, 1.0)	.14
Cesarean section, *n*(%)	115 (29%)	11 (31%)	1.1 (0.5,2.3)	.79	116 (30%)	10 (28%)	0.9 (0.4–1.9)	.81
Gestational age, median (IQR)	37 (36,39)	36 (36,37)	0.8 (0.6, 1.0)	.07	37 (36,39)	36 (36,38)	0.8 (0.7, 1.0)	.15
Allergic rhinitis, *n* (%)	31 (8%)	8 (23%)	3.4 (1.4, 8.2)	< .01	31 (8%)	8 (22%)	3.3 (1.3, 7.8)	< .01
Eczema, *n* (%)	87 (22%)	15 (44%)	2.7 (1.3, 5.6)	< .01	86 (22%)	16 (44%)	2.8 (1.3, 5.6)	< .01
Family history of asthma, *n* (%)	80 (20%)	21 (60%)	5.8 (2.8, 12.0)	< .01	80 (20%)	21 (58%)	5.4 (2.6,11.0)	< .01
Family history of atopic diseases, *n*(%)	150 (38%)	14 (40%)	1.0 (0.5–2.1)	.83	148 (37%)	16 (44%)	1.3 (0.6–2.6)	.43
Passive smoke exposure, *n* (%)	51 (14%)	8 (24%)	1.9 (0.8–4.5)	.11	51 (14%)	8 (24%)	1.8 (0.8–4.3)	.13
Maternal smoking,^b^*n* (%)	25 (6%)	8 (23%)	4.4 (1.8, 10.7)	< .01	25 (7%)	8 (23%)	4.2 (1.7,10.3)	< .01
Childcare attendance,^c^*n* (%)	165 (42%)	18 (51%)	1.4 (0.7, 2.9)	.28	164 (42%)	19 (53%)	1.5 (0.7, 3.0)	.21
Breastfeeding, *n* (%)	327 (85%)	27 (82%)	0.8 (0.3, 2.0)	.66	325 (84%)	29 (85%)	1.0 (0.3, 2.8)	.89

^a^Age at the last follow-up date

^b^maternal smoking status during pregnancy

^c^Childcare attendance before 3 years

^d^Unadjusted Odds ratio

## Discussion

Our study results suggest that developing an NLP algorithm for API mining from EHR was feasible, as demonstrated by both criterion and construct validity. Our NLP-API algorithm has a potential to overcome the current challenges for asthma ascertainment in asthma care and research, enabling large-scale asthma studies by identifying children who meet API, current asthma guideline-recommended criteria [24].

Our study results show that the NLP-API asthma status was highly correlated with manual chart review, and this concordance was not affected by gender, ethnicity, or gestational age, suggesting criterion validity (Table 3). The study findings suggest 88% positive predictive value and 99% negative predictive value for ascertaining asthma. Discrepancies between NLP-API and manual chart review were in part because 1) the abstractors reviewed *parental* records for parental history of asthma, but NLP used only child’s medical records (e.g., the note section of Family history), and 2) NLP often misinterpreted “cold” in “wheezing without cold” although both NLP and human abstractor used the pre-defined definition of “cold” [22, 23]. Also, the associations of known risk factors for asthma (e.g., maternal smoking during pregnancy) with the NLP-API determined asthma status were similar to those determined by manual chart review, suggesting construct validity. In contrast, the widely used method of ascertaining asthma status such as ICD-9 codes showed poor sensitivity as noted in our previous study—sensitivity of ICD codes was 31% whereas the NLP had 97% sensitivity although different asthma criteria was used [16, 28]. Studies based on self-reporting of asthma status such as questionnaire and survey data are subject to significant misclassification bias. For example, almost a quarter of parents whose children were admitted to the hospital for asthma did not report a history of asthma in their children [11]. There have been studies based on ascertaining asthma status with the help of lab tests (e.g., eosinophils) or biomarkers for asthma ascertainment [29], but these tests are impractical when a large number of patients or large study cohorts are involved. Importantly, our study results suggest that the NLP algorithm for API not only ascertains asthma status but also identifies associated individual risk factors such as a family history of asthma, allergic rhinitis, and atopic dermatitis which are part of API [30].

EHRs have been around since the 1960s when they were introduced as a technique to guide and teach medical professionals about how to handle medical knowledge [31]. In the early twenty-first century, a need for standardizing national data, as transmitting health information across organizational and regional boundaries was brought forth [32]. The Health Information Technology for Economic and Clinical Health (HITECH) Act of 2009 directed the Office of the National Coordinator for Health Information Technology (ONC) to promote the adoption and meaningful use of electronic health records. By 2014, 3 out of 4 (76%) hospitals had adopted at least a basic EHR system [33], and there are growing trends of applying EHR to clinical and translational research. For example, clinical studies are facilitating investigators in identifying the characteristics of patients and discovering phenotypes from EHR such as Electronic Medical Records and Genomics (eMERGE) [34]. NLP algorithms have been used for automated encoding of text data into structured data [35], extraction of molecular pathways from articles [36], translation of information from chest radiographs [37] as well as identification of medical complications in postoperative patients from EHR [38]. Thus, given the growing worldwide deployment of EHR systems and the usefulness of free-text embedded in medical records in capturing text-based events, an NLP algorithm will be an important tool to overcome the current challenges of processing large-scale data in disease ascertainment or phenotypic characterization.

Indeed, in our previous studies [16–18], we were able to establish the application of NLP algorithms for asthma ascertainment using an existing other asthma criteria, and to our knowledge, this was the first exploration to apply NLP algorithms to asthma ascertainment as other algorithms using NLP for asthma were for identifying physician diagnosis of asthma or asthma guideline, but not for applying existing asthma criteria [39–41]. This current study is an extension of our previous work by adding an NLP algorithm for API. Given the lack of gold standard to ascertain asthma status, National Asthma Education and Prevention Program guidelines suggest using API for asthma management. API has been used for prospective as well as retrospective studies [22, 42]. As EHRs are likely to continue to be used in clinical research described above, an NLP algorithm for API can be beneficial in the future.

Our study results have several implications in clinical practice, research, and public health. In clinical practice, it enables clinicians and health care systems to apply NLP-API for population management strategies. Using NLP-API, health care systems may identify children who meet API during early childhood (e.g., < 3 years old) on a regular basis for their better access to preventive and therapeutic interventions for asthma, and temporal and geographic trends of outcomes to these interventions may be assessed and monitored at a population level. The impact of a delay in diagnosis of asthma on asthma outcomes can also be examined. Allocation of resources could then be guided by this surveillance system. In research, while NLP-API addresses the limitations of the current methods of asthma ascertainment, it is an innovative approach enabling large-scale clinical studies minimizing methodological heterogeneity of asthma ascertainment due to human biases and mistakes when they review medical records, especially for a large volume of patients. There is a noteworthy finding in our error analysis to examine discrepancies of patient’s API status between NLP-API and manual chart review. Independent third reviewer (an allergist) assessed the discrepancies, and we found that the NLP-API was able to capture API-positive patients that were missed by manual chart review as humans could miss or overlook asthma-related events during the review of large volumes of medical records. Thus, the NLP approach might potentially open up a venue to improve or correct human errors in processing a large volume of data or text review, and this needs to be further studied. Use of a computer based algorithm for ascertaining asthma becomes helpful in public health surveillance as it allows health care systems to monitor the trends of asthma prevalence and incidence in real-time and assess the impact of asthma on serious health outcomes (e.g., susceptibility to serious and common infections including vaccine preventable diseases) [21].

The main strength of our study is the epidemiological advantages of conducting retrospective studies in a study setting that is virtually a self-contained health care environment. In addition, under the auspices of the Rochester Epidemiology Project, we were able to capture all inpatient and outpatient asthma-related events for this present study from birth to the last follow-up date [26]. Our NLP algorithm has a unique capability to determine asthma index (inception) date, which helps researchers discern temporality [22]. An additional strength of this study is the unique aspect of NLP algorithm incorporating free-text data (e.g., asthma symptoms), lab data (e.g., eosinophil count), and structured data (e.g., self-reported response collected at clinic visit).

Limitations of our study includes a retrospective study design with a relatively small sample size, and thus, we were not able to fully address the associations between asthma status and certain risk factors such as second-hand smoking exposure and breastfeeding history. Although it was not statistically significant, NLP-API still showed strong associations with the expected direction for these factors. Another potential limitation is the portability of applying NLP-API to different EHR and health care systems. Appropriate adjustments to NLP algorithms may be necessary to address the intrinsic heterogeneity of EHR in order to produce a desirable performance of NLP algorithms at a different study setting. While our previous study demonstrated the portability of NLP asthma ascertainment tool based on a different asthma criteria using EHR [18, 43], the study result of NLP-API needs to be validated in different clinical settings to ensure the portability. In addition, there were the difficulties of semantic understanding of complex assertion status in clinical narratives (e.g., identifying asthma-related concepts that are negated, hypothetical, or associated with other family members), resulting in false negatives and false positives. Intrinsic limitation of EHR reliability collected from the sources may not represent complete history of patient’s conditions (eg, parents’ asthma collected from family history section and patient provided information) and thus affect the final asthma ascertainment. Our earlier work based on a prospective cohort study showed a close correlation between medical events (mild acute illnesses) in EMR and those captured by prospective follow up [44, 45]. Lastly, our cohorts used for the training (Mayo Clinic sick child care cohort) and test (preterm-weighted cohort) from a single center may not represent general population in children.

## Conclusion

In conclusion, our NLP-API algorithm may prove to be valuable not only in the research realm where it can aid with large-scale clinical studies, but it also has the ability to help the clinician as a population management tool as well becoming a method of surveillance for the public health sector.

## Abbreviations

API: Asthma Predictive Index
EHR: Electronic Health Record
ICD: International Classification of Diseases
NIH: National Institute of Health
NLP: Natural Language Processing
